# PTEN‐AKT pathway attenuates apoptosis and adverse remodeling in ponatinib‐induced skeletal muscle toxicity following BMP‐7 treatment

**DOI:** 10.14814/phy2.15629

**Published:** 2023-03-22

**Authors:** Ayushi Srivastava, Dinender K. Singla

**Affiliations:** ^1^ Division of Metabolic and Cardiovascular Sciences, Burnett School of Biomedical Sciences College of Medicine, University of Central Florida Orlando Florida USA

**Keywords:** apoptosis, atrophy, cell death, skeletal muscle myopathy, tyrosine kinase inhibitor

## Abstract

Tyrosine kinase inhibitors (TKIs) including ponatinib are commonly used to treat cancer patients. Unfortunately, TKIs induce cardiac as well as skeletal muscle dysfunction as a side effect. Therefore, detailed mechanistic studies are required to understand its pathogenesis and to develop a therapeutic treatment. The current study was undertaken to examine whether ponatinib induces apoptosis and apoptotic mechanisms both in vitro and in vivo models and furthermore to test the potential of bone morphogenetic protein 7 (BMP‐7) as a possible treatment option for its attenuation. Sol8 cells, a mouse myogenic cell line was exposed to ponatinib to generate an apoptotic cell culture model and were subsequently treated with BMP‐7 to understand its protective effects. For the in vivo model, C57BL/6J mice were administered with ponatinib to understand apoptosis, cell signaling apoptotic mechanisms, and adverse muscle remodeling and its attenuation with BMP‐7. TUNEL staining, immunohistochemistry (IHC), and real‐time polymerase chain reaction (RT‐PCR) methods were used. Our data show significantly (*p* < 0.05) increased TUNEL staining, caspase‐3, BAX/Bcl2 ratio in the in vitro model. Furthermore, our in vivo muscle data show ponatinib‐induced muscle myopathy, and loss in muscle function. The observed muscle myopathy was associated with increased apoptosis, caspase‐3 staining, and BAX/Bcl‐2 ratio as confirmed with IHC and RT‐PCR. Furthermore, our data show a significant (*p* < 0.05) increase in the involvement of cell signaling apoptotic regulator protein PTEN and a decrease in cell survival protein AKT. These results suggest that increased apoptosis following ponatinib treatment showed an increase in skeletal muscle remodeling, sarcopenia, and fibrosis. Furthermore, BMP‐7 treatment significantly (*p* < 0.05) attenuated ponatinib‐induced apoptosis, BAX/Bcl2 ratio, decreased PTEN, and increased cell survival protein AKT, decreased adverse muscle remodeling, and improved muscle function. Overall, we provide evidence that ponatinib‐induces apoptosis leading to sarcopenia and muscle myopathy with decreased function which was attenuated by BMP‐7.

## Graphical abstract

BMP‐7 attenuates ponatinib‐induced muscle toxicity via PTEN‐AKT pathway. The figure describes the mechanisms of ponatinib‐induced muscle toxicity.

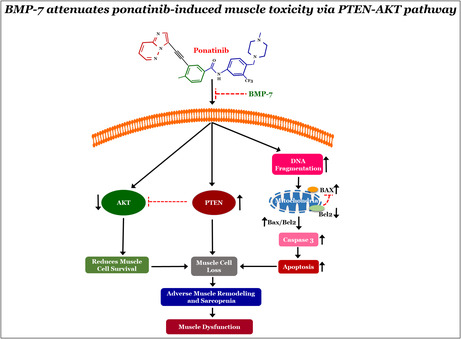

## 1 INTRODUCTION

Ponatinib is a third generation tyrosine kinase inhibitor (TKI), used for various types of cancer therapies such as leukemia, lung cancer, and glioblastoma (Gozgit et al., 2012; Luciano et al., 2020; Tan et al., 2019; Zhang et al., 2014). However, clinical utilization of TKIs is associated with several adverse toxicities in various organs including cardiovascular, hepatic, and gastrointestinal (Bauer et al., 2016; Shah et al., 2013). Ponatinib‐induced cardiotoxicity has recently been shown to activate apoptosis in cardiomyocytes and causes cardiac dysfunction (Casavecchia et al., 2020; Chan et al., 2020; Ma et al., 2020; Singh et al., 2019; Talbert et al., 2015). Additionally, loss of skeletal muscle mass and muscle myopathy due to TKIs is a commonly recognized adverse drug reaction (Rinninella et al., 2020, 2021; Uchikawa et al., 2020), seen in around 80% of chronic myeloid leukemia patients (Janssen et al., 2019). The musculoskeletal symptoms reported in pre‐clinical trials of ponatinib and other TKI studies include muscular cramps, myalgia, pain, and fatigue (Bouitbir et al., 2020; Cortes et al., 2012, 2018; Janssen et al., 2019, 2020; Kekäle et al., 2015). These symptoms have debilitating effects on disease control and impair the quality of life in patients (Janssen et al., 2019, 2020). However, much is unknown about the effect of ponatinib on skeletal muscle cells and the resulting muscle myotoxicity.

Apoptosis is a type of programmed cell death that maintains tissue homeostasis during development and aging (Elmore, 2007). The early stage of apoptosis includes inducing signals for death receptors, increased reactive oxygen species, and change in protein levels of BCL2‐Associated X Protein (BAX), B‐cell lymphoma 2 (Bcl2), and BCL2‐associated agonist of cell death (BAD), this is followed by activation of mitochondrial pathway and commitment to cell death (Elmore, 2007; Shamas‐Din et al., 2013). Hallmarks for apoptosis include caspase‐mediated protein cleavage, chromatin condensation, and DNA fragmentation (Liu & Ahearn, 2001). Dysregulation of apoptosis is known to play important role in multiple diseased conditions including cardiac infarction, neurological disorders, diabetes, cancer, and muscular dystrophy (Anversa et al., 1998; Reed, 1999; Savitz & Rosenbaum, 1998; Serdaroglu et al., 2002). Apoptosis has also been associated with skeletal muscle atrophy (Dirks & Leeuwenburgh, 2005; Dupont‐Versteegden, 2006; Schwartz, 2008). However, the lack of understanding of the exact cause of apoptosis and its mechanisms in ponatinib‐induced muscle myopathy is still unknown.

Available therapeutic strategies for skeletal muscle toxicity following TKI treatment have been recommended to patients, however, evidence‐based research, treatment efficacy, and pharmacological interactions have not yet been established. Thus, alternate therapeutic strategies are needed. Bone morphogenetic protein 7 (BMP‐7) is an anti‐inflammatory growth factor belonging to the transforming growth factor beta superfamily which plays an important role in various biological processes (Aluganti Narasimhulu & Singla, 2020). Previous studies have established that BMP‐7 has anti‐fibrotic, anti‐apoptotic, and anti‐inflammatory effects in cardiomyocytes of infarcted hearts and diabetic cardiomyopathy (Aluganti Narasimhulu & Singla, 2020, 2021; Elmadbouh & Singla, 2021; Urbina & Singla, 2014). It remains unknown whether BMP‐7 could be a potential candidate to attenuate ponatinib‐induced skeletal muscle toxicity.

Therefore, in the current study, to the best of our knowledge, we establish for the first time: (1) ponatinib induces apoptosis in skeletal muscle cells both in vitro and in vivo models, (2) ponatinib‐induced apoptosis is mediated through BAX‐Bcl2 and PTEN‐Akt pathway, (3) Increased apoptosis enhances skeletal muscle fibrosis and remodeling, (4) BMP‐7 treatment inhibits apoptosis, BAX‐Bcl2 ratio, PTEN‐Akt pathway, and skeletal muscle adverse remodeling, (5) Most importantly, BMP‐7 treatment improves skeletal muscle function.

## 2 MATERIALS AND METHODS

### 2.1 Cell culture model

Sol8 cells (mouse myogenic cell line) were obtained from the American Type Culture Collection (ATCC) and maintained in Dulbecco's Modified Eagle's Medium (DMEM; ThermoFisher Scientific; cat# 11965092), as we reported previously (Tavakoli Dargani et al., 2018). The DMEM medium was supplemented with 10% fetal bovine serum (R&D Systems; cat# S11550), glutamine (ThermoFisher Scientific; cat# 25030081), sodium pyruvate (ThermoFisher Scientific; cat#11360070), and penicillin–streptomycin (P/S; ThermoFisher Scientific; cat# 15070063), and cells were cultured at 37°C, 5% CO_2_.

### 2.2 MTT assay

The ponatinib dose curve was established via the MTT assay. A 96‐well plate was used to culture the Sol8 cells that were treated with an increasing concentration of ponatinib (Selleck Chemicals; cat# S1490) (4, 8, 10, 12, 14, and 16 μm) to determine the optimal concentration and cell viability. MTT Kit (Roche; cat# 11465007001) was used as per the instructions provided by the manufacturer. Briefly, mitochondrial activity was assessed by the formation of formazan crystals as an indicator of cell viability. The optical density of the solubilized purple formazan crystals was measured at 550 and 600 nm via Bio‐Rad plate reader (Bio‐Rad) (Singla, Garner, et al., 2019). Cell viability data were presented as a percentage of the control values.

### 2.3 BrdU assay

The effect of ponatinib and BMP‐7 on cell proliferation was determined by BrdU Cell Proliferation enzyme‐linked immunosorbent assay (ELISA) Kit (Abcam; cat# ab126556). Sol8 cells were plated in a 96‐well plate (10^4^ cells/well) and treated with 8 μM ponatinib and 500 ng/mL of BMP‐7 (Bioclone; cat# PA‐0401) for 24 h, which was then replaced with fresh DMEM for 24 h. Manufacturer's protocol provided with the kit was used to perform the cell proliferation assay. In brief, BrdU reagent (20 μL/well) was added to the cells for 24 h. Cells were fixed with fixing solution (200 μL/well, 30 min, room temperature [RT]) and washed as per the instructions followed by incubation with anti‐BrdU detector antibody (100 μL/well, 1 h, RT). Peroxidase Goat Anti‐Mouse IgG conjugate (100 μL/well, 30 min, RT) was added, followed by final wash with both wash buffer and distilled water. Finally, the 3,3′,5,5′‐tetramethylbenzidine peroxidase substrate (100 μL/well, 30 min, RT) was added followed by the addition of Stop solution (100 μL/well). Absorbance was measured at 450 and 550 nm via Bio‐Rad plate reader and net absorbance was calculated by subtracting the blank absorbance (Bhansali et al., 2021).

### 2.4 Preparation of apoptotic cell culture model

Sol8 cells were cultured for 24 h in a 96‐well plate or in 8‐well plates (10^4^ cells/well). Three different study groups were assigned: control, ponatinib, and ponatinib + BMP‐7. Cells were treated with 8 μM of ponatinib (24 h) and 500 ng/mL of BMP‐7 (24 h), followed by replacement with DMEM for 24 h.

### 2.5 Animal preparation and experimental design

The Institutional Animal Care and Use Committee (IACUC) and The University of Central Florida (UCF) approved all the animal procedures and protocols. A total of 43 C57BL/6J male and female mice (10 ± 2‐week‐age) (JAX) were split into three groups (*n* = 14–15 animals/group): control (0.9% saline), ponatinib (5 mg/kg/day; cumulative dose of 25 mg/kg body weight) and ponatinib (5 mg/kg/day) + BMP‐7 (200 μg/kg/day; cumulative dose of 600 μg/kg body weight). Saline and ponatinib were administered for five consecutive days (Monday to Friday) via intraperitoneal injection and BMP‐7 via intravenous injection on three alternative days (Monday, Wednesday, and Friday). The dose for ponatinib and BMP‐7 used in the present study was followed as previously reported (Aluganti Narasimhulu & Singla, 2021; Zhang et al., 2014). Body weights were recorded prior to the first injection (initial weight) and on the day of the sacrifice (final weight). On day 14 (D‐14) mice were subjected to muscle function tests followed by euthanization via cervical dislocation under 4% isoflurane. Blood samples and bilateral soleus muscle (SM) tissues were collected after mice sacrifice. SM tissues were washed with 1× phosphate buffer saline (PBS), weighed, and stored at −80°C for RNA or saved in 4% paraformaldehyde (PFA) for immunohistochemical (IHC) staining, ELISA, and histological staining.

### 2.6 Body weight and muscle function analysis

#### 2.6.1 Body and SM weight

Change in body weight was evaluated by subtracting the initial body weight recorded prior to any treatment administration from the final weight recorded on the day of the sacrifice (D‐14). Ratio of SM weight to body weight was calculated to determine the change in SM mass after ponatinib administration.

#### 2.6.2 Grip strength test

A grip strength meter (Columbus Instruments) was used to measure the grip strength of both the forelimbs and four limbs (combined) as mentioned previously (Aluganti Narasimhulu & Singla, 2021). In brief, the mouse was held and allowed to grab the grid by forelimbs and then by combined four‐limbs and gently pulled away in a horizontal manner. The grip strength was recorded in real‐time as peak force in grams. Six to nine trials were averaged and normalized with body weight (g). SigmaPlot software (Systat Software, Inc.) was used to plot the bar graphs.

#### 2.6.3 Weights test

Muscle strength was further assessed using weights test as reported previously (Aluganti Narasimhulu & Singla, 2021; Dessouki et al., 2020). Briefly, mice were subjected to carry weights in increasing order from 15 to 65 g (15, 25, 35, 45, 50, 55, 60, and 65 g) for 3 s. Analysis was done by calculating two scores: weigh × time (WT) and trial × time (TT). TT was scored by multiplying the trial number by the time of hold and WT was scored by multiplying the highest weight held by the time the weight was held. Data was presented by bar graph plotted using SigmaPlot software.

### 2.7 Tissue processing and deparaffinization

After 48 h in 4% PFA, SM tissues were washed with PBS thrice and transferred to 70% ethanol. The tissues were processed using Leica TP1020 tissue processing system (Leica Biosystems). Tissue Tek TEC (Sakura Finetek) was used to embed SM tissues and sectioned at 5 μm thickness using a Microm HM 325 (Fisher Scientific) and placed on microscopic slides (Aluganti Narasimhulu & Singla, 2022).

### 2.8 TUNEL staining

Apoptosis was determined using TUNEL staining for both Sol8 cells and SM tissue sections. In Situ Cell Death Detection Kit (TMR red, Roche; cat# 12156792910) was used according to the provided protocol and as previously described (Singla & McDonald, 2007). Cells were washed, dried, and directly mounted with Antifade Vectashield mounting medium containing 4′,6‐diamidino‐2‐phenylindole (DAPI; Vector laboratories; cat# H‐120010) to stain the nuclei.

SM sections were deparaffinized using xylene, following rehydration by successive incubation in decreasing concentration of ethanol (100%, 90%, 70%, 50%, 30%) and a final wash with distilled water. Tissue sections were pre‐treated with Proteinase K (25 μg/mL in 100 mM Tris–HCL) and Tunel assay was performed as mentioned in Merino and Singla (2018) followed by blocking with 10% normal goat serum (NGS, Vector Laboratories; cat# S‐1000) and further co‐stained with Myosin (1:100; Sigma Aldrich; cat# M7523‐1ML) followed by secondary antibody, (Alexa 488 goat anti‐rabbit, 1:200; ThermoFisher Scientific; cat# A11008). Sections were then washed with PBS and covered with DAPI‐containing mounting medium. The percentage of apoptotic nuclei was calculated by dividing the total TUNEL positive nuclei by total DAPI positive nuclei [(total TUNEL^+ve^/total DAPI) × 100]. Four areas were imaged using a Keyence BZ‐X810 fluorescent microscope (Keyence), and quantified using ImageJ. Fluorescence and Brightfield representative images were taken in ×20 and ×40 magnification and histograms were made using Sigma Plot.

### 2.9 Immunofluorescence staining

For immunocytochemistry (ICC) staining, Sol8 cells (10^4^ cells/well) were treated, fixed permeabilized, and blocked (10% NGS) as described previously (Tavakoli Dargani et al., 2018). Double Immunohistochemistry (IHC) staining was performed on SM tissue sections as previously published (Aluganti Narasimhulu & Singla, 2021). Briefly, SM sections underwent deparaffinization, rehydration, and blocking by 10% NGS. Prior to staining with apoptotic markers SM tissue sections were co‐stained with Myosin (overnight at 4°C) and Alexa 488 goat anti‐rabbit (1:200; 1 h 30 m at RT). Both cells and tissue sections were incubated with primary antibodies of pro‐apoptotic markers: caspase 3 (1:300; Santa Cruz Biotechnology; Cat# sc‐7148), BAX (1:300; Santa Cruz Biotechnology; Cat# sc‐493) and anti‐apoptotic marker Bcl2 (1:300; Santa Cruz Biotechnology; Cat# sc‐492) overnight at 4°C followed by washing with PBS and incubation with secondary antibody, (Alexa 568 goat anti‐rabbit; 1:1000 for in vitro and 1:500 for in vivo; Invitrogen; cat# A11011) for 1 h 30 min at RT. Finally, after washing nuclei were stained by Vectashield mounting medium with DAPI (Aluganti Narasimhulu & Singla, 2022; Dessouki et al., 2020). Images were recorded and quantified as previously described (Aluganti Narasimhulu & Singla, 2021; Singla, Johnson, et al., 2019). BAX to Bcl2 ratio was also calculated by dividing the percent of BAX‐positive cells by the percent of Bcl2‐positive cells (Salakou et al., 2007).

### 2.10 Real‐time polymerase chain reaction analysis

For real‐time polymerase chain reaction (RT‐PCR), SM tissue was used to isolate RNA by TRIzol™ Reagent (ThermoFisher; cat# 15596018) following cDNA synthesis using SuperScript™ III First‐Strand Synthesis SuperMix for quantitative real‐time polymerase chain reaction (qRT‐PCR, ThermoFisher; cat# 11752050). qRT‐PCR was performed by CFX96 C1000 Touch™ Thermal Cycler Multicolor Real‐Time PCR Detection System (Bio‐Rad) with SYBR Green (Invitrogen; cat# 11761500). PCR was performed using mouse primers (Table 1) for: caspase 3, BAX, Bcl2, PTEN, and AKT for apoptotic marker gene expressions and glyceraldehyde 3‐phosphate dehydrogenase as a loading control. Melt curves were established for the reactions and normalized fold expressions were calculated using the 2^−ΔΔCT^ method (Aluganti Narasimhulu & Singla, 2022).

**TABLE 1:** Mouse‐specific primers used for the study.

Target	Forward primer	Reverse primer
AKT	5′‐ATCCCCTCAACAACTTCTCAGT‐3′	5′‐CTTCCGTCCACTCTTCTCTTTC‐3′
BAX	5′‐CTGGATCCAAGACCAGGGTG‐3′	5′‐CTTCCAGATGGTGAGCGAGG‐3′
BCL2	5′‐GAACTGGGGGAGGATTGTGG‐3′	5′‐GCATGCTGGGGCCATATAGT‐3′
Caspase‐3	5′‐GAGCTTGGAACGGTACGCTA‐3′	5′‐GAGTCCACTGACTTGCTCCC‐3′
GAPDH	5′‐ACCCAGAAGACTGTGGATGG‐3′	5′‐CACATTGGGGGTAGGAACAC‐3′
PTEN	5′‐CATTGCCTGTGTGTGGTGATA‐3′	5′‐AGGTTTCCTCTGGTCCTGGTA‐3′

### 2.11 Enzyme‐linked immunosorbent assay

For ELISA Assay 25 μg of protein extracted from SM tissue homogenate (Merino & Singla, 2018) was used as per the provided instructions for Mouse Phosphatase and Tensin Homologs (PTEN; MYBIOSOURCE; cat# MBS3806814) and Mouse RAC‐alpha serine/threonine (AKT; MYBIOSOURCE; cat# MBS288382) ELISA kits. Absorbance was measured at 450 nm using Bio‐Rad plate reader and histograms for each were plotted as arbitrary units.

### 2.12 Histological staining

Hematoxylin and eosin (H&E) staining was performed on SM sections as described previously (Aluganti Narasimhulu & Singla, 2021). The muscle sections are stained pink whereas nuclei are stained blue/purple. Myofibrillar size and atrophy were quantified by assessing the myocyte size (mm^2^). For Masson's staining, interstitial fibrosis (IF) was determined in SM sections as previously published (Singla, Johnson, et al., 2019). SM sections were stained red, nuclei in black, and fibrosis in blue. IF was measured by quantifying the total fibrotic area (in blue) in mm^2^. After staining sections were mounted with permount and three to four areas/section were recorded using light microscopy under Keyence BZ‐X810 for quantification at 20× magnification via ImageJ. Representative images were taken at 40× magnification.

### 2.13 Statistical analysis

Statistical significance was analyzed between groups using Student's *t*‐test and one‐way analysis of variance followed by Tukey test using Sigma Plot. All values are presented as ±standard error of mean, with *p* < 0.05 considered statistically significant.

## 3 RESULTS

### 3.1 Effect of ponatinib on skeletal muscle cell viability

**FIGURE 1:**
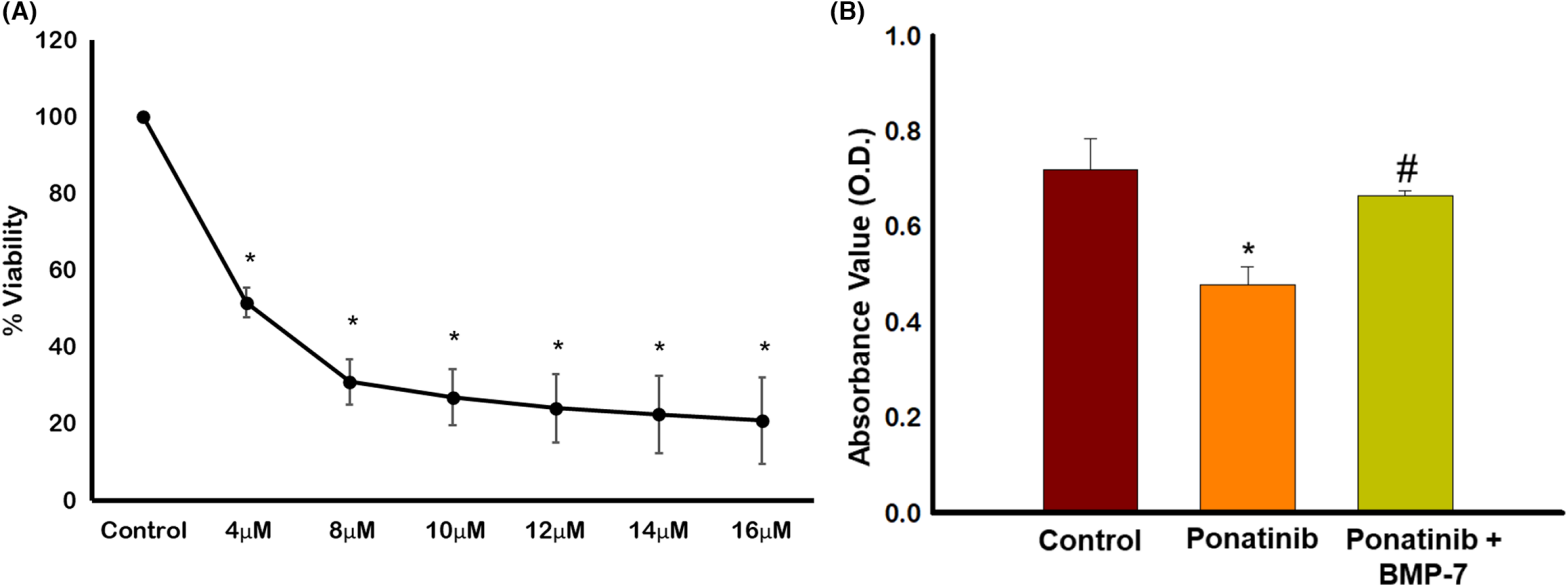
Effect of ponatinib on soleus muscle (Sol8) cell viability and BMP‐7 on cell proliferation. Representative graph shows (A) dose‐dependent toxicity of ponatinib on Sol8 cells, units presented in % viability (*n* = 6; in quadruplicates); (B) quantitative analysis of the effect of ponatinib and BMP‐7 on Sol8 cell proliferation, units presented in optical density (*n* = 6–12; in duplicates). Error bar = ±standard error of the mean. Statistical significance was analyzed using Student's *t*‐test and One‐way ANOVA followed by Tukey test. *p < 0.05 versus Control; ^#^ *p* < 0.05 versus ponatinib. ANOVA, analysis of variance; BMP‐7, bone morphogenetic protein 7.

To evaluate dose‐dependent toxicity of ponatinib on SM (Sol8) cells, MTT assay was performed. As shown in Figure 1A ponatinib doses from 4 to 16 μM significantly (*p* < 0.05) decreased cell viability, with increased concentration of ponatinib as compared to control. Based on these results, further in vitro experiments, were performed using 8 μM ponatinib concentration.

### 3.2 Effect of BMP‐7 treatment on skeletal muscle cells proliferation

To further determine the effects of ponatinib on cell proliferation, BrdU cell proliferation ELISA assay was performed. Figure 1B shows a significant reduction in cell proliferation (*p* < 0.05) by ponatinib when compared to control. We observed a significant (*p* < 0.05) increase in Sol8 cell proliferation in ponatinib + BMP‐7 group compared to the ponatinib group‐suggesting BMP‐7 plays a protective role in cell proliferation following ponatinib treatment.

### 3.3 Effect of BMP‐7 treatment on ponatinib‐induced apoptosis and pro‐apoptotic marker caspase 3 in Sol8 cells

**FIGURE 2:**
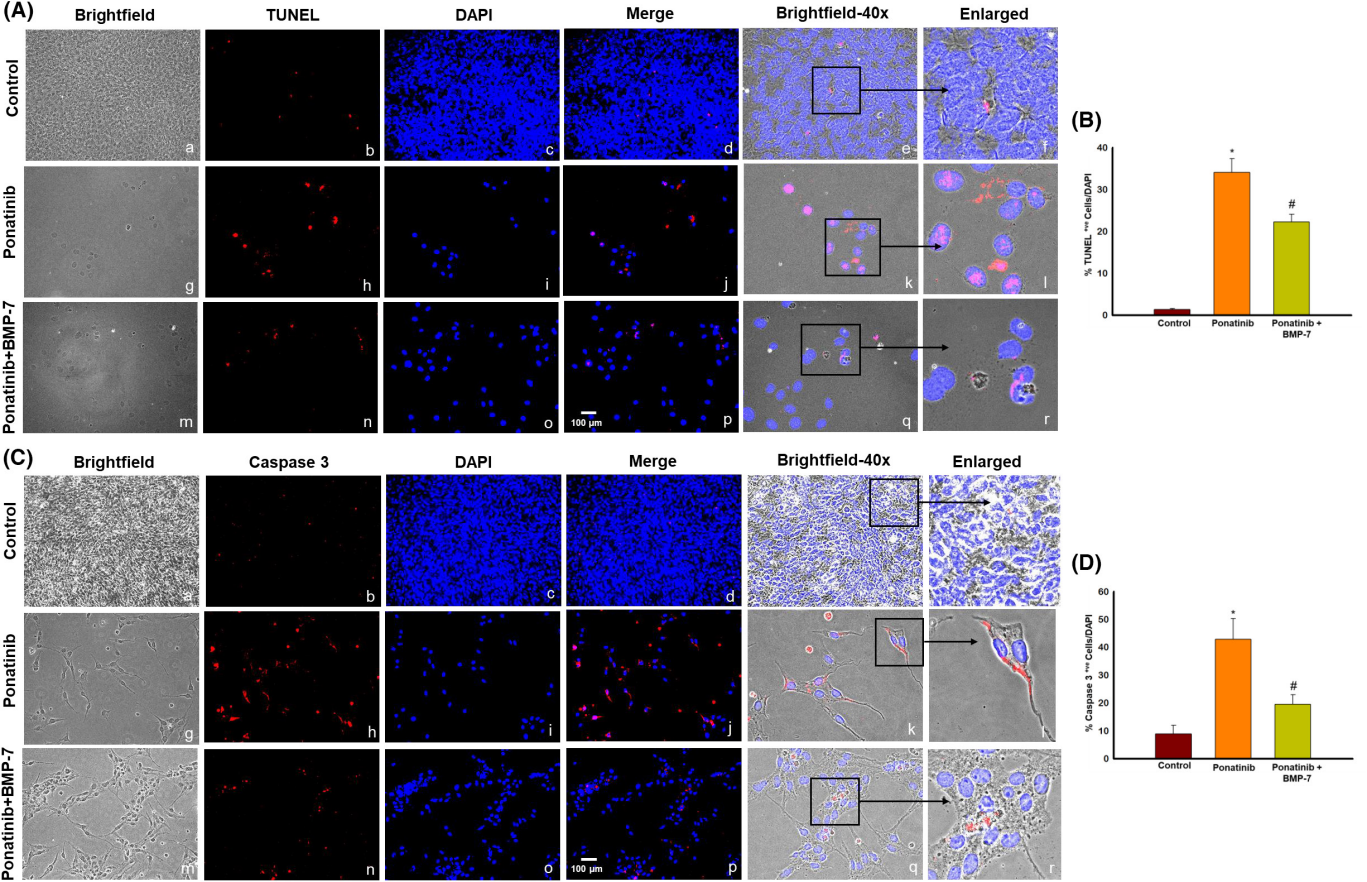
Effect of BMP‐7 treatment on ponatinib‐induced apoptosis and pro‐apoptotic marker caspase 3 in Sol8 cells. Representative images show positive cells for (A) TUNEL staining and (C) caspase 3. (A, C) Show (×20) brightfield images (a, g, m), TUNEL and caspase 3‐positive cells in red (b, h, n), DAPI in blue (c, i, o), and merged images (d, j, p). Scale bar = 100 μm. ×40 Brightfield merged images (e, k, q) and black boxes and arrows indicate enlarged sections of brightfield merged images (f, l, r). Histograms (B, D) show quantitative analysis for TUNEL and caspase 3‐positive cells over DAPI (*n* = 4; in triplicates). Error bar = ±standard error of the mean. Statistical significance was analyzed using Student's *t*‐test and One‐way ANOVA followed by Tukey test. *p < 0.05 versus Control; ^#^ *p* < 0.05 versus ponatinib. ANOVA, analysis of variance; BMP‐7, bone morphogenetic protein 7; DAPI, 4′,6‐diamidino‐2‐phenylindole.

TUNEL staining was performed in Sol8 cells to establish ponatinib‐induced apoptosis in Sol8 cells. Figure 2A show brightfield images (Figure 2A[a,g,m], ×20) of Sol8 cells with and without treatment groups of ponatinib and BMP‐7, TUNEL‐positive cells in red (Figure 2A[b,h,n]), total nuclei stained with DAPI in blue (Figure 2A[c,i,o]), merged (Figure 2A[d,j,p], 20×), merged brightfield (Figure 2A[e,k,q], 40×), and enlarged images (Figure 2A[f,l,r]) shows TUNEL‐positive nuclei in pink, suggesting an increase in apoptotic TUNEL nuclei in ponatinib‐treated group as compared to control (Figure 2A[a–f]) and its reduction in the BMP‐7‐treated group. Furthermore, the quantitative analysis in Figure 2B shows a significant increase (*p* < 0.05) in TUNEL‐positive nuclei in ponatinib treated group as compared to control. Whereas significant decrease (*p* < 0.05) in TUNEL‐positive nuclei were observed following BMP‐7 treatment suggesting that BMP‐7 attenuates ponatinib‐induced apoptosis.

Next, to strengthen the finding of TUNEL staining, we performed caspase 3 ICC. Our data show higher number of positive cells for pro‐apoptotic marker caspase 3 in ponatinib (Figure 2C[g–l]) group as compared to control (Figure 2C) whereas ponatinib + BMP‐7‐treated (Figure 2C) group shows decrease in caspase 3 staining. The quantitative data Figure 2D show a significant increase (*p* < 0.05) of caspase 3 in ponatinib‐treated cells as compared to control. Whereas, following BMP‐7 treatment a significant decrease (*p* < 0.05) in caspase 3 staining was observed, suggesting BMP‐7 inhibits caspase 3 expression following ponatinib treatment.

### 3.4 Effect of BMP‐7 treatment on pro‐apoptotic marker BAX, anti‐apoptotic marker Bcl2, and BAX/Bcl2 ratio in Sol8 cells

**FIGURE 3:**
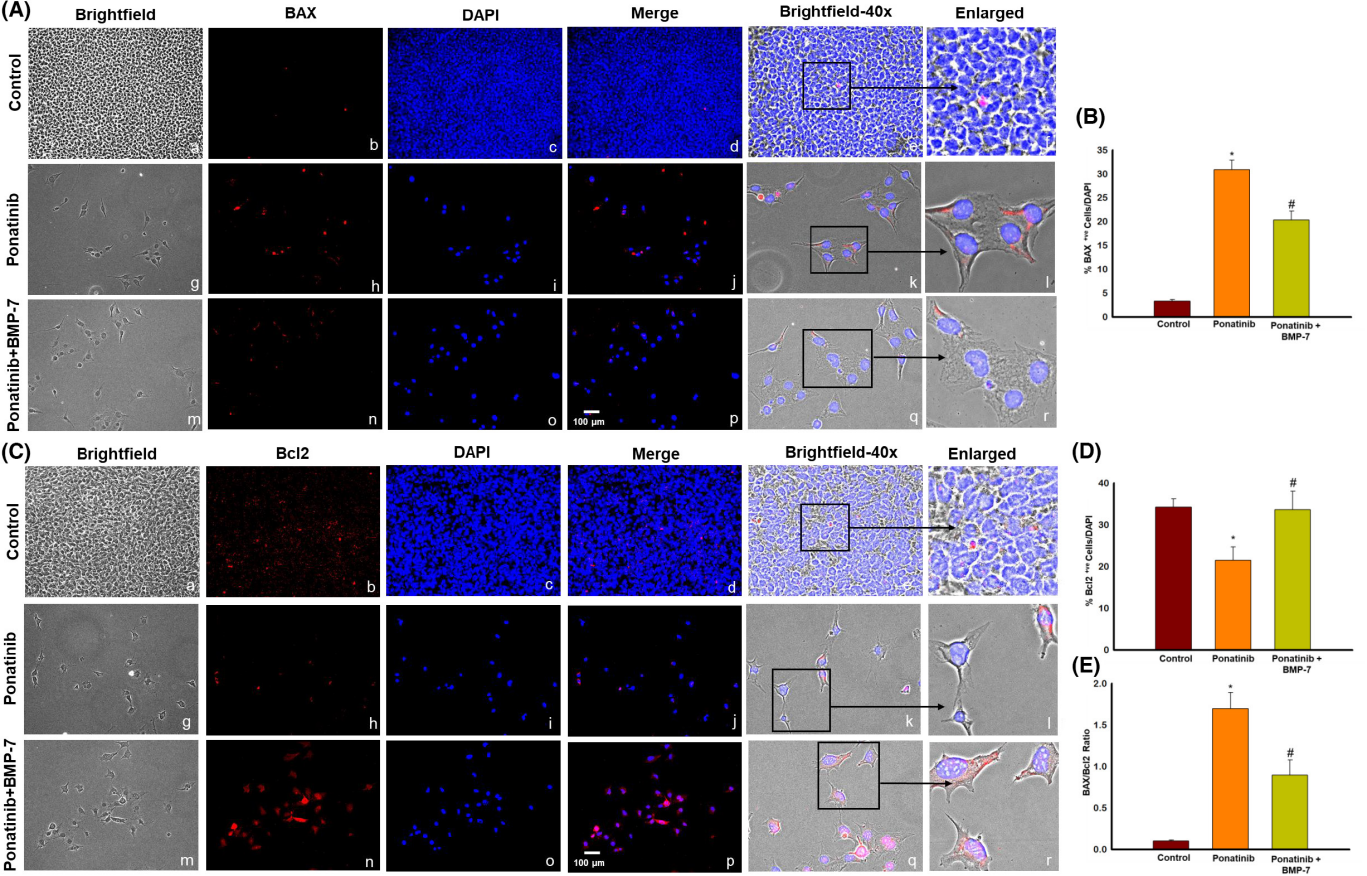
Effect of BMP‐7 treatment on pro‐apoptotic marker BAX, anti‐apoptotic marker Bcl2 and BAX/Bcl2 ratio in Sol8 cells. Representative images show positive cells for (A) pro‐apoptotic marker BAX and (C) anti‐apoptotic marker Bcl2. (A, C) Show (×20) brightfield images (a, g, m), BAX and Bcl2‐positive cells in red (b, h, n), DAPI in blue (c, i, o), merged images (d, j, p). Scale bar = 100 μm. ×40 brightfield merged images (e, k, q) and black boxes and arrows indicate enlarged sections of brightfield merged images (f, l, r). Bar graphs (B, D) show quantitative analysis for pro‐apoptotic marker BAX and anti‐apoptotic marker Bcl2 ICC (*n* = 4; in triplicates). Histogram (E) show the quantitative analysis for BAX/Bcl2 ratio for ICC. Error bar = ±standard error of the mean. Statistical significance was analyzed using Student's *t*‐test and one‐way ANOVA followed by Tukey test. *p < 0.05 versus Control; ^#^ *p* < 0.05 versus ponatinib. ANOVA, analysis of variance; BAX, BCL2‐associated X protein; Bcl2, B‐cell lymphoma 2; BMP‐7, bone morphogenetic protein 7; ICC, immunocytochemistry.

To determine whether ponatinib‐induced apoptosis triggers pro‐apoptotic marker BAX and anti‐apoptotic marker Bcl2 double IHC staining was performed. Our ICC data demonstrate an increase in expression of BAX protein positive cells in ponatinib group compared to control (Figure 3A). We observed a decreased expression of BAX protein following BMP‐7 treatment (Figure 3A). Additionally, our quantitative data analysis shows a significant (*p* < 0.05) increase in BAX staining in ponatinib group as compared to control. Upon BMP‐7 treatment a significant (*p* < 0.05) reduction of BAX positive cells was seen (Figure 3B).

Figure 3C demonstrates the decrease in Bcl2‐positive cells in ponatinib group as compared to controls. Following BMP‐7 treatment Bcl2‐positive cells were increased (Figure 3C). The quantitative data showed a significant (*p* < 0.05) reduction in number of positive cells of Bcl2 in ponatinib group as compared to control. Whereas a significant (*p* < 0.05) increase was observed in ponatinib + BMP‐7 group (Figure 3D). We further investigated the BAX/Bcl2 ratio after ponatinib and BMP‐7 treatment, Figure 3E histogram shows a significant (*p* < 0.05) increase in BAX/Bcl2 ratio in ponatinib group as compared to control and this increase was reversed in BMP‐7 group. This data is further indicative that ponatinib‐induced apoptosis involves BAX and Bcl2 cell signaling.

### 3.5 Effect of BMP‐7 treatment on ponatinib‐induced weight loss and sarcopenia

**FIGURE 4:**
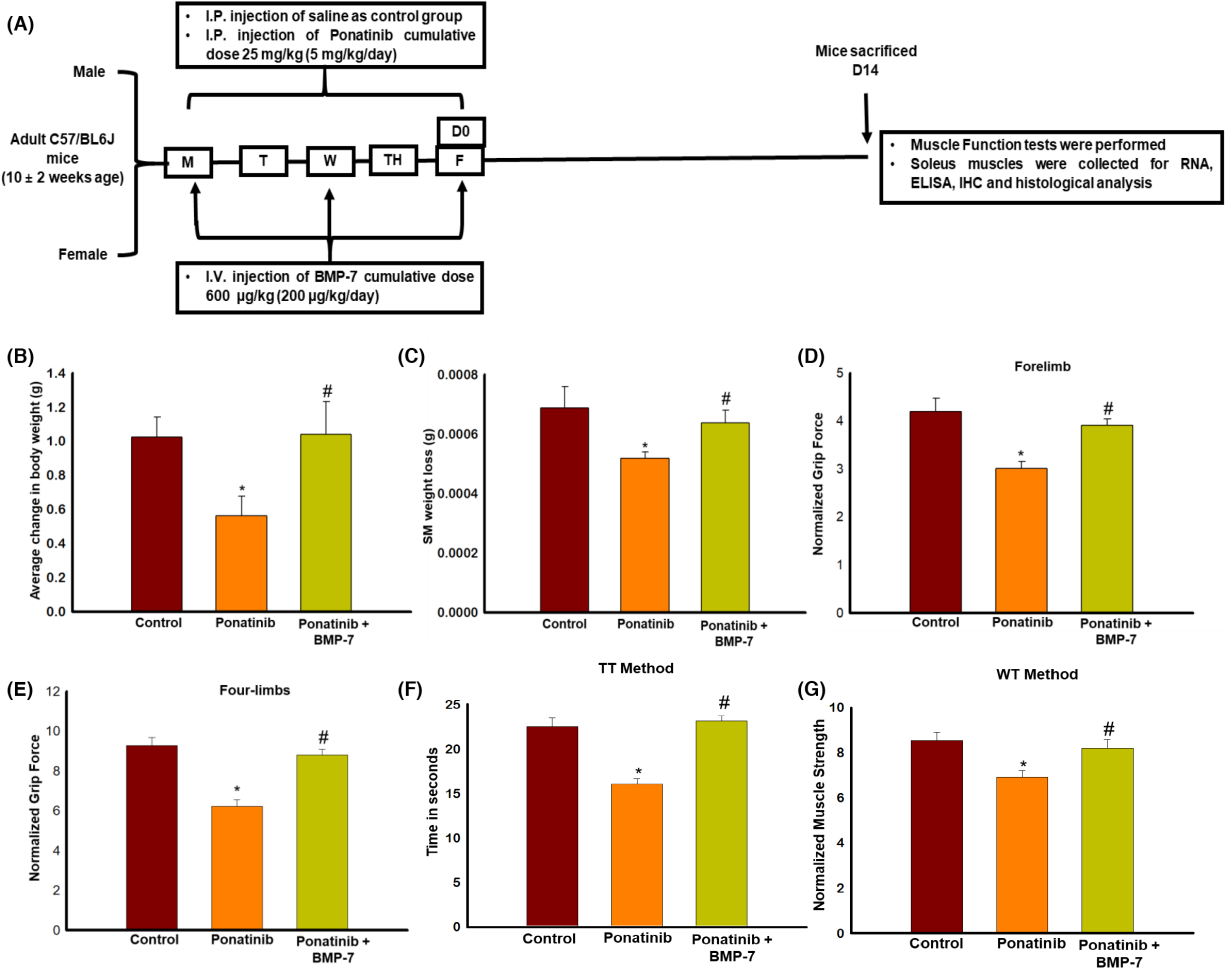
Effect of BMP‐7 treatment on ponatinib‐induced weight loss, sarcopenia and muscle dysfunction. (A) Schematic representation of the injection schedule and the study design. Bar graphs showing increase in (B) body weight (BW) (C) soleus muscle weight on BMP‐7 administration. Muscle function was evaluated on day 14 before mice sacrifice. Bar graphs representing the quantification and analysis for multiple muscle function tests (D) forelimb grip strength, (E) four‐limbs grip strength, (F) weight's test‐TT Test and (G) weight's test‐WT test (*n* = 14–15). Error bar = ±standard error of the mean. Statistical significance was analyzed using Student's *t*‐test and one‐way ANOVA followed by Tukey test. *p < 0.05 versus control; ^#^ *p* < 0.05 versus ponatinib. ANOVA, analysis of variance; BMP‐7, bone morphogenetic protein 7; TT, trial × time; WT, weigh × time.

Change in body weight after ponatinib and BMP‐7 administration was assessed by measuring weight gain and weight loss across the three groups. Figure 4B shows a significant (*p* < 0.05) loss in weight after ponatinib administration as compared to control and a significant (*p* < 0.05) weight gain was noted upon BMP‐7 treatment. We further investigated the effect of ponatinib and BMP‐7 on SM mass loss. Change in SM weight to body weight was calculated. Figure 4C shows ponatinib‐treated muscles had significantly (*p* < 0.05) reduced weight and developed sarcopenia as compared to control. Whereas a significant (*p* < 0.05) improvement in SM mass was seen after the BMP‐7 treatment. This is indicative of BMP‐7 being a potential therapeutic intervention that can be administered to attenuate weight loss and sarcopenia exhibited after ponatinib treatment.

### 3.6 Effect of BMP‐7 treatment on ponatinib‐induced muscle dysfunction

To study the effect of ponatinib on the loss of muscle function and assess its improvement by BMP‐7, mice were subjected to the following tests: (1) grip strength for forelimbs and four limbs and (2) weights test. Data for grip strength for both forelimb (Figure 4D) and four limbs (Figure 4E) show a significant (*p* < 0.05) loss of grip strength in the muscles of ponatinib‐treated mice as compared to control. However, following BMP‐7 treatment a significant (*p* < 0.05) improvement was noticed in the grip strength tests. Weights test data was achieved by analyzing TT method and the WT method. For both methods, as depicted in Figure 4F,G, a significant (*p* < 0.05) decrease in the forelimb muscle strength was observed in ponatinib mice as compared to control, which was significantly (*p* < 0.05) improved upon BMP‐7 treatment group. This is suggestive that BMP‐7 treatment improves ponatinib‐induced muscle dysfunction.

### 3.7 Effect of BMP‐7 treatment on ponatinib‐induced apoptosis and pro‐apoptotic marker caspase 3 in SM

**FIGURE 5:**
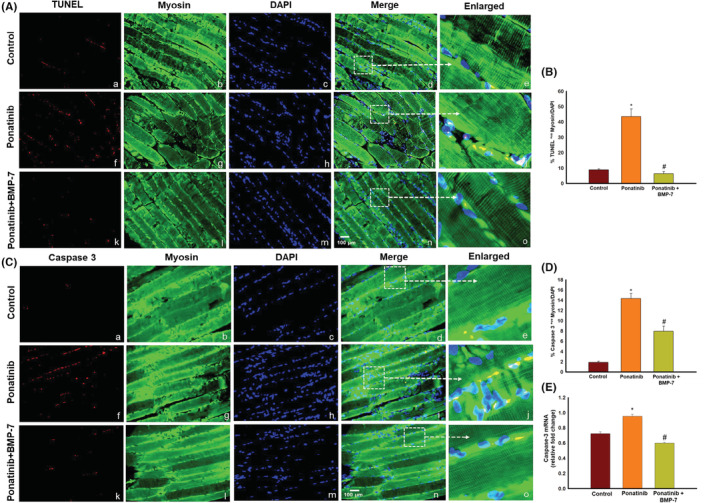
Effect of BMP‐7 treatment on ponatinib‐induced apoptosis and pro‐apoptotic marker caspase 3 in SM tissue. As shown in ×40 representative photomicrographs of SM tissue sections (A) TUNEL staining and (C) caspase 3 demonstrating positive cells stained red (a, f, k), myosin stained muscle cells in green (b, g, l), total nuclei stained in blue with DAPI (c, h, m) and merged images (d, i, n). Scale bar = 100 μm. Dotted white boxes and arrows indicate enlarged sections of merged images (e, j, o). Bar graphs (B, D) represent quantitative analysis of IHC (*n* = 7–8) and graph (E) show the gene expression for caspase 3 (*n* = 4–6). Error bar = ± standard error of the mean. Statistical significance was analyzed using Student's *t*‐test and one‐way ANOVA followed by Tukey test. *p < 0.05 versus control; ^#^ *p* < 0.05 versus ponatinib. ANOVA, analysis of variance; BMP‐7, bone morphogenetic protein 7; DAPI, 4′,6‐diamidino‐2‐phenylindole; IHC, immunohistochemistry; SM, soleus muscle.

To further confirm our in vitro findings of ponatinib‐induced apoptosis, we performed TUNEL staining to assess ponatinib‐induced apoptosis and its attenuation by BMP‐7 on SM tissue sections. We demonstrated TUNEL‐positive nuclei (Figure 5A) and caspase 3‐positive cells (Figure 5C). The images show a significantly higher number of TUNEL‐positive nuclei in ponatinib‐treated SM (Figure 5A[f–j]) in comparison with control (Figure 5A[a–e]) whereas a decrease in TUNEL‐positive cells was noticed in ponatinib + BMP‐7 group (Figure 5A[k–o]). Furthermore, the quantitative analysis shows a significant (*p* < 0.05) increase in TUNEL‐positive nuclei in ponatinib‐treated group as compared to control. However, the ponatinib + BMP‐7 group had a significant decrease in TUNEL‐positive nuclei following BMP‐7 treatment as compared to the ponatinib group (Figure 5B).

Pro‐apoptotic marker caspase 3 was further examined by double IHC. Our data show increased expression of pro‐apoptotic marker caspase 3 in ponatinib group as compared to control. Whereas a decrease in caspase 3‐positive cells is noticeably seen following BMP‐7 treatment (Figure 5C). Next, the quantitative data show a significant increase (*p* < 0.05) of caspase 3 in ponatinib‐treated group as compared to control and its significant decrease (*p* < 0.05) after BMP‐7 treatment (Figure 5D). To strengthen our IHC findings, RT‐PCR analysis was performed, which shows a significant increase (*p* < 0.05) in caspase 3 gene expression in ponatinib‐treated mice as compared to control (Figure 5E). After BMP‐7 administration a significant (*p* < 0.05) reduction in caspase 3 gene expression was observed. Both the in vitro and in vivo studies coincide in confirming the occurrence of apoptosis induced by ponatinib which is attenuated following BMP‐7 treatment.

### 3.8 Effect of BMP‐7 treatment on pro‐apoptotic marker BAX, anti‐apoptotic marker Bcl2, and BAX/Bcl2 ratio in SM

**FIGURE 6:**
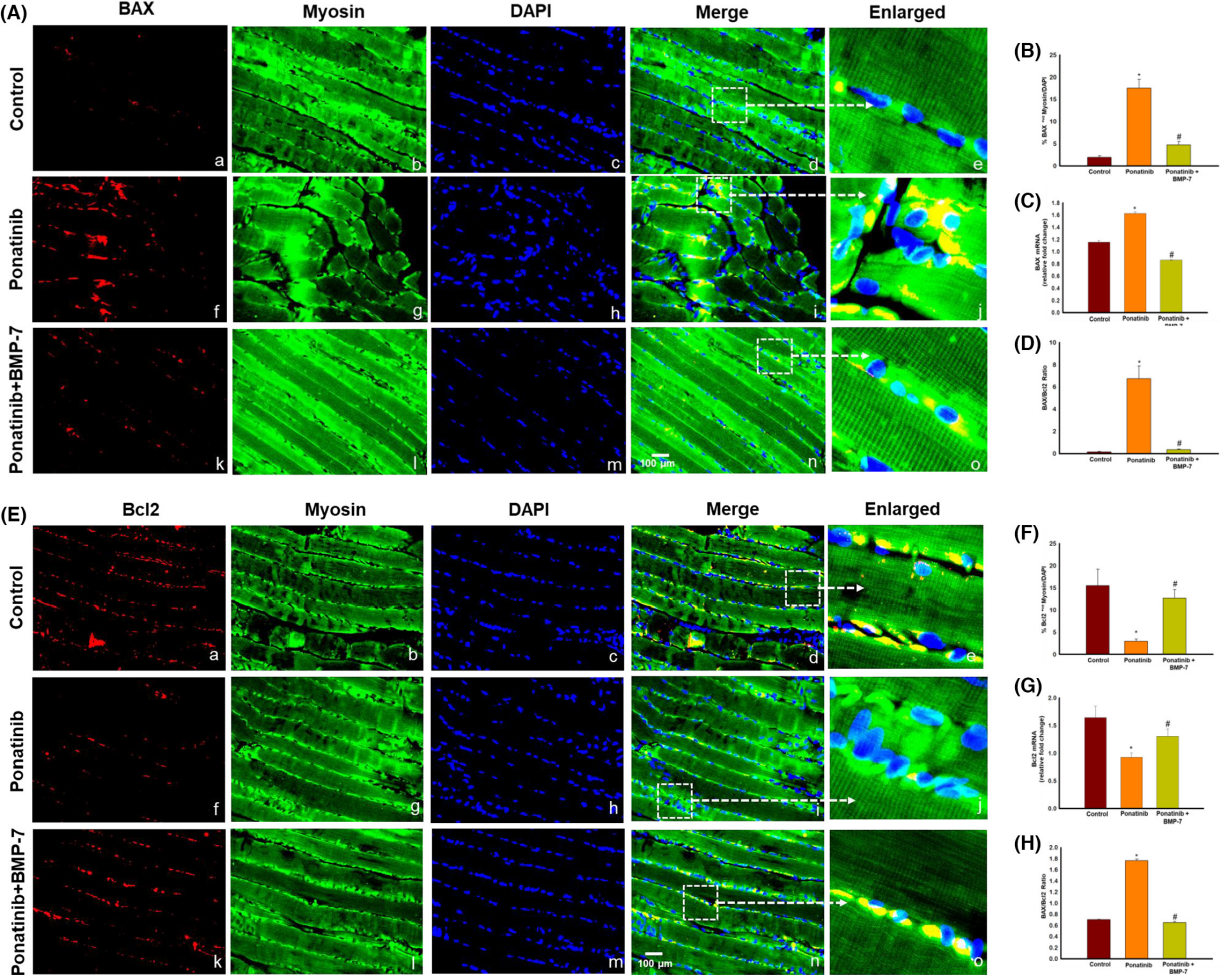
Effect of BMP‐7 treatment on pro‐apoptotic marker BAX, anti‐apoptotic marker Bcl2 and BAX/Bcl2 ratio in SM tissue. As shown in ×40 representative photomicrographs of SM tissue sections (A) BAX and (D) Bcl2‐positive cells are stained red (a, f, k), muscle cells stained green by myosin (b, g, l), DAPI stained nuclei in blue (c, h, m) and merged images (d, i, n). Scale bar = 100 μm. Dotted white boxes and arrows indicate enlarged sections of merged images (e, j, o). Bar graphs (B, E) represent quantitative analysis of IHC (*n* = 7–8) and graphs (C, F) show the gene expression for BAX (*n* = 5–6) and Bcl2 (*n* = 5–7). Histograms (G, H) show the quantitative analysis for BAX/Bcl2 ratio for IHC and real‐time polymerase chain reaction. Error bar = ±standard error of the mean. Statistical significance was analyzed using Student's *t*‐test and one‐way ANOVA followed by Tukey test. *p < 0.05 versus control; ^#^ *p* < 0.05 versus ponatinib. ANOVA, analysis of variance; BAX, BCL2‐associated X protein; Bcl2, B‐cell lymphoma 2; BMP‐7, bone morphogenetic protein 7; DAPI, 4′,6‐diamidino‐2‐phenylindole; IHC, immunohistochemistry; SM, soleus muscle.

The effect of ponatinib and BMP‐7 on pro‐apoptotic marker BAX and anti‐apoptotic marker Bcl2 was further determined by IHC and RT‐PCR. Our IHC data show BAX‐positive cells are increased in ponatinib (f‐j, Figure 6A) group as compared to control (Figure 6A[a–e]). Following BMP‐7 administration BAX‐positive cells decreased in number (Figure 6A[k–o]). Our quantitative data further demonstrates a significant (*p* < 0.05) increase in BAX‐positive cells in ponatinib group as compared to control and its significant (*p* < 0.05) attenuation in ponatinib + BMP‐7 group (Figure 6B). RT‐PCR data analysis for BAX gene showed a significant (*p* < 0.05) higher expression following ponatinib treatment as compared to control; however, a significant (*p* < 0.05) reduction in BAX gene expression was observed after BMP‐7 administration (Figure 6C).

Our anti‐apoptotic data show decreased number of Bcl2‐positive cells in ponatinib (Figure 6E[f–j]) group as compared to control (Figure 6E[a–e]). Noticeably, an increase was seen in Bcl2 following BMP‐7 treatment (Figure 6E[k–o]). The quantitative data also supports a significant (*p* < 0.05) reduction in number of positive cells of Bcl2 in ponatinib group as compared to control whereas a significant (*p* < 0.05) increase was observed in ponatinib + BMP‐7 group (Figure 6F). RT‐PCR data analysis in Figure 6G shows significantly (*p* < 0.05) lowered Bcl2 gene expression in ponatinib group as compared to control, whereas the Bcl2 gene expression significantly (*p* < 0.05) increased in BMP‐7 treated mice. Next, we evaluated the BAX/Bcl2 ratio using both IHC and RT‐PCR data. The quantitative analysis in Figure 6D,H shows a significant (*p* < 0.05) increase in BAX/Bcl2 ratio in ponatinib group as compared to control and ponatinib + BMP‐7 groups. This set of data strengthens the initial data of the in vitro study in our in vivo model, suggesting that significant changes in BAX, Bcl2 expression, and BAX/Bcl2 ratio occur due to ponatinib‐induced apoptosis. Furthermore, indicating BMP‐7 administration ameliorates pro‐apoptotic marker BAX and BAX/Bcl2 ratio and promotes anti‐apoptotic Bcl2 expression.

### 3.9 Effect of BMP‐7 treatment on cell signaling markers PTEN and AKT

**FIGURE 7:**
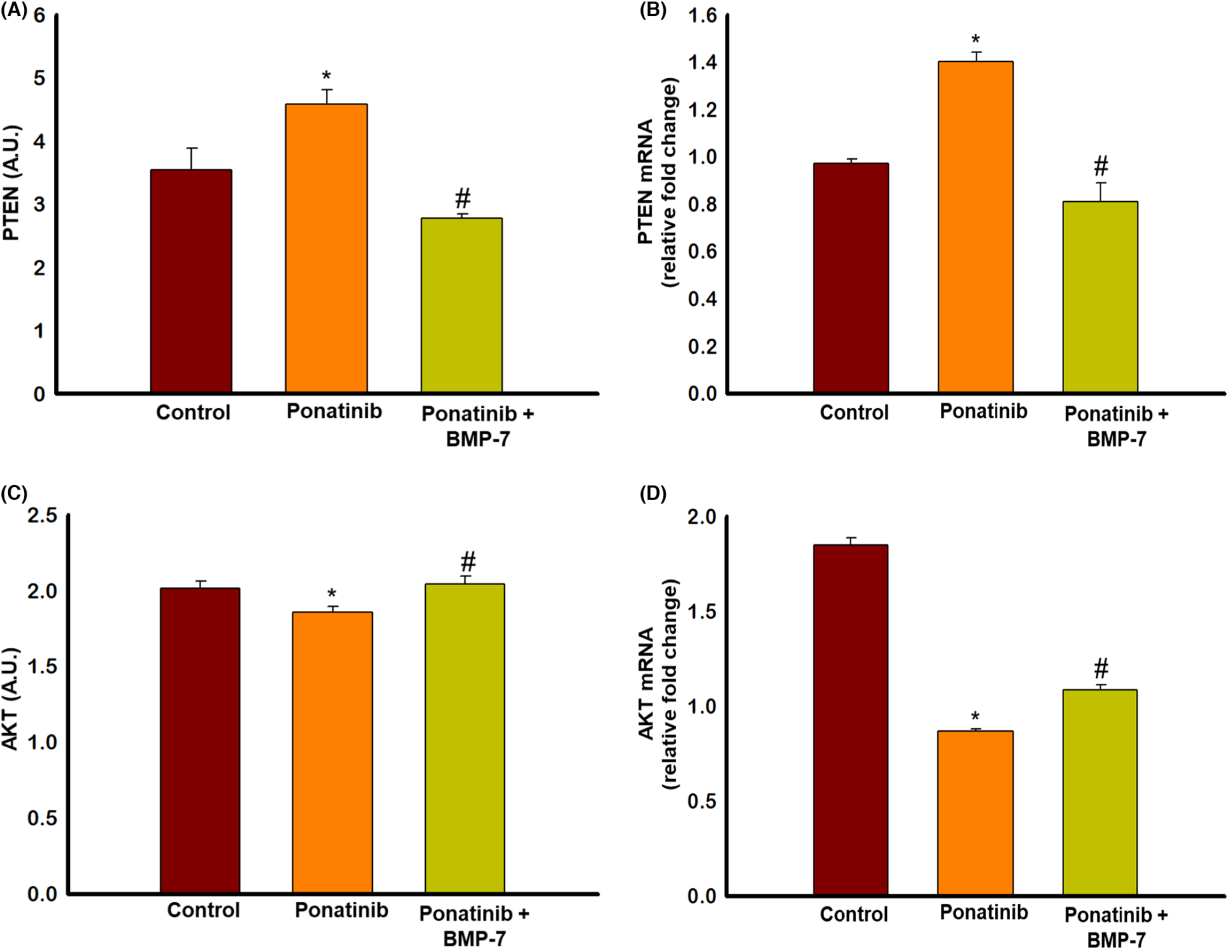
Effect of BMP‐7 treatment on cell signaling markers PTEN and AKT. Enzyme‐linked immunosorbent assay and real‐time polymerase chain reaction was performed using SM tissue to determine the levels of PTEN and AKT. Histograms (A, B) represent the quantitative analysis of PTEN and AKT levels in 25 μg of protein (*n* = 6–8). Bar graphs (C, D) show the gene expression for PTEN (*n* = 5–6) and AKT (*n* = 5–6). Error bar = ±standard error of the mean. Statistical significance was analyzed using Student's *t*‐test and one‐way ANOVA followed by Tukey test. *p < 0.05 versus control; ^#^ *p* < 0.05 versus ponatinib. ANOVA, analysis of variance; BMP‐7, bone morphogenetic protein 7; SM, soleus muscle.

To determine the regulatory effect of ponatinib and BMP‐7 on pro‐apoptotic regulator of apoptosis protein PTEN and pro‐survival protein AKT, we examined expressions of both PTEN and AKT with ELISA and RT‐PCR. Our ELISA data show a significant (*p* < 0.05) increase in PTEN expression of ponatinib‐treated SM, compared to control (Figure 7A). However, a significant (*p* < 0.05) reduction in expression was observed following BMP‐7 treatment (Figure 7A). This data was further confirmed using PTEN gene expression where we observed a significant increase of PTEN in ponatinib which was decreased following BMP‐7 treatment. However, our ELISA data on pro‐survival protein AKT demonstrate significant (*p* < 0.05) decrease in expression levels in the ponatinib group when compared to control and its significant (*p* < 0.05) increase in ponatinib + BMP‐7‐treated group (Figure 7C). RT‐PCR data following the pattern of AKT ELISA data and support its role in the regulation of ponatinib‐induced apoptosis. This set of data suggests that ponatinib regulates ponatinib‐induced apoptosis through PTEN‐AKT pathway which is attenuated by BMP‐7.

### 3.10 Effect of BMP‐7 treatment on ponatinib‐induced muscle atrophy and adverse muscle remodeling

**FIGURE 8:**
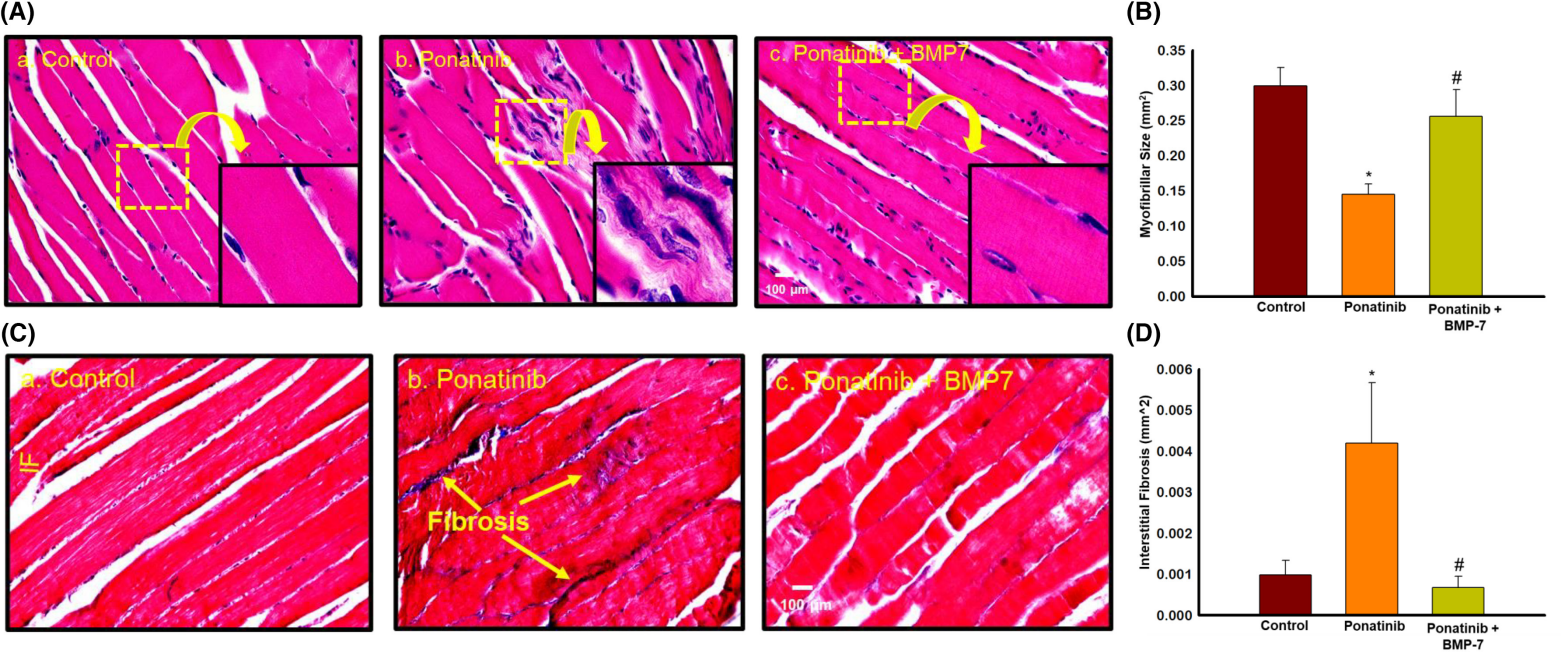
Effect of BMP‐7 treatment on ponatinib‐induced muscle atrophy and adverse muscle remodeling. Representative images (×40) of SM sections (A) stained with hematoxylin and eosin to detect atrophy and (C) of Masson's trichrome staining to show interstitial fibrosis in soleus muscle on day 14 after ponatinib administration in control and experimental groups. Stained sections were quantified at ×20 magnification and magnified for visualization of atrophy and interstitial fibrosis. Bar graphs (B, D) represent quantitative analysis for muscle atrophy and interstitial fibrosis (*n* = 6–8). Error bar = ±standard error of the mean. Statistical significance was analyzed using Student's *t*‐test and one‐way ANOVA followed by Tukey test. *p < 0.05 versus control; ^#^ *p* < 0.05 versus ponatinib. Scale bar = 100 μm. ANOVA, analysis of variance; BMP‐7, bone morphogenetic protein 7; SM, soleus muscle.

To evaluate if ponatinib‐induced apoptosis causes muscle atrophy and adverse muscle remodeling and to determine their attenuation with BMP‐7. H&E and Masson's trichrome staining were performed on SM tissue sections. In Figure 8A, representative photomicrographs of H&E staining show a visible decrease in the myofibrillar size of SM tissue in the ponatinib‐treated group (Figure 8A,B) as compared to control (Figure 8A[a]) suggesting atrophy. Whereas in ponatinib + BMP‐7‐treated group following BMP‐7 administration (Figure 8A[a–c]) a significant increase in the muscle cell size was observed as compared to ponatinib‐treated SM. Quantitative analysis in Figure 8B confirmed that ponatinib‐treated SM tissues had significantly (*p* < 0.05) reduced muscle cell size as compared to control. Whereas BMP‐7 administered mice showed a significant (*p* < 0.05) increase in their muscle cell size.

Skeletal muscle fibrosis in ponatinib mice and its reduction by BMP‐7 was assessed by Masson's Trichrome staining. Representative photomicrographs in Figure 8C show increased collagen (blue area) in ponatinib‐treated SM tissues (Figure 8C[b]) as compared to control (Figure 8C[a]) indicating IF. Following BMP‐7 treatment a significant decrease in IF was observed (Figure 8C[c]) as compared to the ponatinib group. The quantitative data in Figure 8D also shows a significant (*p* < 0.05) increase in IF in ponatinib administered mice as compared to control and its significant (*p* < 0.05) decrease in BMP‐7‐treated mice.

## 4 DISCUSSION

Ponatinib is a potent orally bioavailable TKI known to induce cardiotoxicity and myotoxicity as major adverse side effects (ClinicalTrials.gov, 2020a, 2020b; Casavecchia et al., 2020; Chan et al., 2020; Ma et al., 2020; Singh et al., 2019; Talbert et al., 2015). Very limited studies have been done on ponatinib till now, Phase 2 pre‐clinical trials of ponatinib have reported muscle spasms, muscular weakness, musculoskeletal pain, and myalgia (ClinicalTrials.gov, 2020a, 2020b). The forenamed adverse side effects justified a study to investigate the mechanism of action for ponatinib‐induced skeletal muscle toxicity. As of now, as per the best of our knowledge there are no published studies on ponatinib muscle toxicity in animals. The current work is a novel study being performed regarding ponatinib‐induced toxicity in SM cells and the protective effect of BMP‐7 treatment in both in vitro and in vivo models. This study presents the following major and important information on ponatinib that demonstrates muscle toxicity as follows: (1) decrease in Sol8 cell viability and proliferation, (2) increase in apoptotic‐positive nuclei as confirmed with TUNEL staining and upregulation of pro‐apoptotic markers caspase 3 and BAX, (3) decrease in anti‐ apoptotic marker Bcl2 expression, (4) increase in PTEN, a negative regulator of apoptosis and decrease in pro‐survival protein AKT, (5) decrease in body weight and sarcopenia development, (6) increase in muscle dysfunction, and finally (7) development of muscle atrophy and adverse muscle remodeling in SM. BMP‐7 treatment attenuates muscle toxicity both in in vitro and in vivo via decreasing apoptosis, decreased pro‐apoptotic markers, increasing levels of anti‐apoptotic proteins, reversing adverse muscle remodeling, and improving muscle function.

First, we identified the correct dose of ponatinib‐induced muscle toxicity (PMIT) in Sol8 cells in vitro using MTT assay. After ponatinib dose confirmation we aimed to understand whether this anti‐cancer drug has any effects on Sol8 cell proliferation. The result of our data demonstrated a significant decrease in cell proliferation following ponatinib treatment. Our data show significant skeletal muscle cell toxicity in Sol8 cells by ponatinib in a dose‐dependent manner which correlates with previously published studies on ponatinib‐induced dose‐dependent cytotoxicity in endothelial cells, cardiomyocytes, and cancer cells (Casavecchia et al., 2020; Gozgit et al., 2011; Liu et al., 2019; Saussele et al., 2020; Singh et al., 2019; Talbert et al., 2015).

Apoptosis is a programmed cell death mechanism identified by DNA fragmentation and mitochondrial‐mediated protein such as caspase 3. Ponatinib‐induced apoptosis in Sol8 cells was initially determined by significant increase in DNA fragmentation using TUNEL staining and increased levels of caspase 3. Furthermore, presence of apoptosis was confirmed by upregulation of pro‐apoptotic protein BAX and downregulation of anti‐apoptotic protein Bcl2 in in vitro studies. To further understand the significance of ponatinib‐induced apoptosis in cell culture model, we developed ponatinib‐induced muscle toxicity in an in vivo model. Our model shows significant decrease in body weight, loss of muscle mass with decreased muscle function. Loss of skeletal muscle mass and body weight during TKI therapy has been associated with dose limiting clinical toxicities which leads to negative clinical outcomes such as temporary or permanent treatment discontinuation and decrease in overall survival of patients (Rinninella et al., 2020, 2021). Therefore, our animal studies are in agreement with clinical studies using ponatinib and other TKIs (ClinicalTrials.gov, 2020a, 2020b; Janssen et al., 2019; Kekäle et al., 2015). Next, we investigated the impact of ponatinib‐induced sarcopenia by assessing the muscle function of mice. A significant increase in muscle dysfunction was observed in both grip strength test and weight's test for ponatinib‐treated mice. This anti‐cancer drug‐induced muscle toxicity model corroborates with other published studies on muscle toxicity (Dessouki et al., 2020; Merino & Singla, 2018).

Additionally, we performed TUNEL staining and caspase 3 staining on SM to confirm our in vitro findings. Our data show significantly increased apoptosis and caspase 3 using immunostaining and RT‐PCR methods. Next, to strengthen our apoptosis findings, we performed Bcl2 and BAX IHC and gene expression. Bcl2 family of interacting proteins play a pro‐survival role whereas family members BAX and Bcl‐2 homologus antagonist/killer (BAK) play an opposing role in the regulation of apoptosis in cancer, cardiomyocyte, and muscle cells (Aluganti Narasimhulu & Singla, 2022; Merino & Singla, 2018; Ramadan et al., 2019). However, the role of BAX and Bcl2 proteins in ponatinib‐induced apoptosis is not well‐established; therefore, our performed studies suggest a significant increase in BAX protein following ponatinib treatment whereas a decrease in Bcl2 protein was observed using IHC and RT‐PCR. Moreover, significant increase in BAX/Bcl2 ratio for both ICC and IHC were noticed. Our data on BAX and Bcl2 agrees with other reported studies published on hypoxia‐induced muscle and cardiac apoptosis (Cho et al., 2004; Webster et al., 1999).

Next, we confirmed whether ponatinib‐induced apoptosis in SM is mediated by PTEN‐AKT pathway. Loss of PTEN due to mutations in cancer cells upregulates cell proliferation mediated by Akt pathway whereas upregulation of PTEN in cells induces apoptosis (Johnson & Singla, 2018; Singla, 2015), therefore, balance on PTEN/Akt confirms cell survival vs cell death. Moreover, PTEN is a negative regulator of pro‐survival protein AKT. Previous studies have shown inhibition of pro‐survival pathways during ponatinib‐induced apoptosis in cardiomyocytes (Merino & Singla, 2018; Singh et al., 2020; Talbert et al., 2015). However, the role of PTEN‐AKT pathway in ponatinib‐induced apoptosis in SM is not known. A significant increase in PTEN expression was observed in ponatinib‐treated SMs. Moreover, a significant decrease in AKT expression was noticed following ponatinib treatment. Our data in the present study suggest that ponatinib‐induced apoptosis in SM is regulated by PTEN‐AKT pathway.

Following apoptosis confirmation in ponatinib‐induced SM, it becomes reasonable to ask whether apoptosis has further effects on muscle remodeling. Therefore, we examine myofibrillar size for muscle atrophy and IF using H&E and Mason's trichrome histological staining. Our data showed significant loss of myofibrillar size and increased collagen deposition in the SM after ponatinib administration, suggesting presence of atrophy and fibrosis. This muscle remodeling data agrees with previously published studies showing apoptosis‐induces atrophy and muscle fibrosis in variety of conditions such as aging, inflammation, and liver cirrhosis (Dirks & Leeuwenburgh, 2005; Kurosawa et al., 2021; Lala‐Tabbert et al., 2019; Saito et al., 2020).

Based on current literature, there are no readily available therapeutic options to treat ponatinib‐induced skeletal muscle toxicity. In this study, we identified BMP‐7 a growth factor belonging to the transforming growth factor‐β superfamily, commonly given to osteoporosis patients as a potential therapeutic agent. BMP‐7 has previously been reported to have anti‐fibrotic properties in heart, muscle, and kidney (Aluganti Narasimhulu & Singla, 2020, 2021; Elmadbouh & Singla, 2021; Urbina & Singla, 2014; Zeisberg et al., 2003). The findings of the current study suggests that BMP‐7 promotes Sol8 cell proliferation, inhibits ponatinib‐induced apoptosis in both in vitro and in vivo models as observed by decrease in TUNEL stained nuclei, pro‐apoptotic markers caspase 3 and BAX, and increase in anti‐apoptotic marker Bcl2 expressions. Furthermore, decrease in BAX/Bcl2 ratio, PTEN expression and increased AKT expression also reinforces that BMP‐7 reduces ponatinib‐induced apoptosis. Moreover, significant decrease in loss of body weight and sarcopenia, improved muscle function and decrease in muscle atrophy and fibrosis after BMP‐7 treatment was observed. This set of data suggests potential therapeutic efficacy of BMP‐7 in attenuation of ponatinib‐induced apoptosis and muscle remodeling. This agrees with previously reported studies showing BMP‐7 improves skeletal muscle fibrosis, atrophy, sarcopenia, and muscle dysfunction in diabetes and atherosclerosis (Aluganti Narasimhulu & Singla, 2020, 2021; Elmadbouh & Singla, 2021; Urbina & Singla, 2014).

In conclusion, to the best of our knowledge we report for the first time that ponatinib induces skeletal muscle toxicity via apoptosis in both in vitro (Sol8 cells) and in vivo (SM) models. Apoptosis is confirmed by TUNEL staining, expression of pro‐apoptotic and anti‐apoptotic markers, caspase 3, BAX and Bcl2, and BAX/Bcl2 ratio. Further expanding on how ponatinib‐induced apoptosis leads to the development and progression of muscle myopathy as seen by increase in sarcopenia, muscle dysfunction, atrophy, and adverse muscle remodeling. Evident attenuation of apoptosis, sarcopenia, muscle dysfunction, atrophy, and adverse muscle remodeling after BMP‐7 treatment is promising. These results shed light on BMP‐7 as a potent therapeutic agent against ponatinib‐induced muscle myopathy.

## AUTHOR CONTRIBUTIONS

Dinender K. Singla designed and supervised the study. Ayushi Srivastava performed the experiments, analyzed data, prepared figures, and drafted the manuscript. Dinender K. Singla revised the manuscript and approved final version of the manuscript.

## FUNDING INFORMATION

This study was supported by National Institute of Diabetes and Digestive and Kidney Diseases (R01DK120866‐01) and National institute of Health (5R01CA221813‐04).

## CONFLICT OF INTEREST STATEMENT

The authors declare no competing interests.

## ETHICS STATEMENT

All the animal procedures and protocols were approved by the Institutional Animal Care and Use Committee (IACUC) and The University of Central Florida (UCF).
